# Budget Impact Analysis of Biosimilar Products in Spain in the Period 2009–2019

**DOI:** 10.3390/ph14040348

**Published:** 2021-04-09

**Authors:** Manuel García-Goñi, Isabel Río-Álvarez, David Carcedo, Alba Villacampa

**Affiliations:** 1 Department of Applied & Structural Economics and History, Faculty of Economics and Business, Complutense University of Madrid, Campus de Somosaguas, Pozuelo de Alarcón, 28223 Madrid, Spain; 2 Spanish Biosimilar Medicines Association, BioSim, 28027 Madrid, Spain; isabeldelrio@biosim.es; 3 Hygeia Consulting S. L., 28046 Madrid, Spain; david.carcedo@hygeiaconsulting.com (D.C.); alba.villacampa@hygeiaconsulting.com (A.V.)

**Keywords:** biologics, biosimilars, budget impact analysis, savings, pharmaceutical spending, cost containment, Spain

## Abstract

Since the first biosimilar medicine, Omnitrope^®^ (active substance somatropin) was approved in 2006, 53 biosimilars have been authorized in Spain. We estimate the budget impact of biosimilars in Spain from the perspective of the National Health System (NHS) over the period between 2009 and 2019. Drug acquisition costs considering commercial discounts at public procurement procedures (hospital tenders) and uptake data for both originator and biosimilar as actual units consumed by the NHS were the two variables considered. Two scenarios were compared: a scenario where no biosimilars are available and the biosimilar scenario where biosimilars are effectively marketed. All molecules exposed to biosimilar competition during this period were included in the analysis. The robustness of the model was tested by conducting multiple sensitivity analyses. From the payer perspective, it is estimated that the savings produced by the adoption of biosimilars would reach EUR 2306 million over 11 years corresponding to the cumulative savings from all biosimilars. Three molecules (infliximab, somatropin and epoetin) account for 60% of the savings. This study provides the first estimation of the financial impact of biosimilars in Spain, considering both the effect of discounts that manufacturers give to hospitals and the growing market share of biosimilars. We estimate that in our last year of data, 2019, the savings derived from the use of biosimilars relative total pharmaceutical spending in Spain is 3.92%. Although more research is needed, our evidence supports the case that biosimilars represent a great opportunity to the sustainability of the NHS through rationalizing pharmaceutical spending and that the full potential of biosimilar-savings has not been achieved yet, as there is a high variability in biosimilar uptake across autonomous regions.

## 1. Introduction

Drug research and development has led to market access for many important therapeutic innovations, and undoubtedly is one of the multiple factors that influence population aging [1]. Powrie-Smith [2] points out how new therapies and vaccines have contributed to the fight against communicable diseases, resulting in a significant reduction in the incidence of viruses such as hepatitis B, as well as in infant mortality. Litchenberg [3,4] shows how those innovations have significantly improved the way health systems treat and care for the sick, increasing life expectancy and quality of life. However, those advances have come with an increase in health spending. In fact, Newhouse et al. [5] and Willeme and Dumont [6] have pointed to the advances in health technology and the therapeutic innovations developed as the most important determinants of such increases in health expenditures, and among those, pharmaceutical spending.

Trends in pharmaceutical markets have raised some concerns about the sustainability of pharmaceutical expenditure [7]. Thus, the focus should be placed on spending efficiency rather on cutting spending, to ensure the maximum return on investment in pharmaceutical products. The global pharmaceutical market will exceed USD 1.5 trillion by 2023, as it is expected to grow at a rate of between 3 and 6% per year. However, this growth will be different in different areas of the world, and in fact, in the five main markets of Europe, this growth is expected to be lower, between 1 and 4% [8]. Those expectations are based on list prices that are exclusive of discounts and rebates paid to governments. This is relevant as a divergence of 1.4 percentage points between expenditure measured at list and net prices was found in a forecast of the pharmaceutical expenditure for the EU5 countries from 2017 to 2021 [9]. Thus, considering the net prices seems critical when estimating the economic impact of any health technology, either in-patent or off-patent medicines, in pharmaceutical expenditure. In any case, a significant part of this increase in pharmaceutical spending is related to the appearance and consolidated use in clinical practice of biological medicines [10], especially for the treatment of chronic and life-threatening diseases such as cancer, multiple sclerosis or rheumatoid arthritis [11]. Many biologic products can actually slow the progress of or even prevent disease [10], but normally present a higher price than that of chemical drugs. In 2018, over 30% of all European drug spending was on biological medicines and this percentage is expected to continue to grow [12].

As in the case of generics with chemical medicines, the loss of exclusivity and the expiry of a patent on innovative biological products, hereinafter referred to as the originator, allows the entry into the market of biosimilar products. The major difference between a generic and a biosimilar is that the natural variability and more complex manufacturing of biological medicines do not allow an exact replication, as is the case with generics. Consequently, biosimilars are subjected to a more comprehensive regulatory pathway to ensure that minor differences do not affect safety or efficacy [13].

As stated by the European Medicines Agency, “a biosimilar is a biological medicine highly similar to another already approved biological medicine” and “biosimilars are approved according to the same standards of pharmaceutical quality, safety and efficacy that apply to all biological medicines” [13]. The European Medicines Agency (EMA) is responsible for evaluating the majority of applications to market biosimilars in the European Union. After the first biosimilar (the recombinant human growth hormone or somatropin) was approved in 2006, 62 biosimilar drugs corresponding to 17 active substances received marketing authorization by EMA as of 31 December 2020 [14]. In Spain, 53 biosimilar drugs for 16 active substances have been authorized for marketing to the same date [15]. However, it is worth noting that the design and implementation of pharmaceutical policies on biosimilars fall within the remit of EU Member States.

Biosimilars are lower cost alternatives of originator biologicals and are expected to bring meaningful budgetary savings to health systems. However, the spending on biosimilars is still very low, at around 1.5% of pharmaceutical expenditure in Europe in 2018 [12]. Unfortunately, there are still not many studies that estimate the savings derived from their use. Simoens et al. [16] published a review of budget impact analyses (BIA) of the use of biosimilars, although only focused mainly on two molecules, infliximab and etanercept. Furthermore, several recent studies have tried to estimate the budget impact of the introduction of recent biosimilars for either one or several molecules, at national or local levels in Italy [17,18,19], the UK [20,21] and Canada [22], including also in Canada a simulation exercise in which different penetration scenarios similar to the OECD average were considered [23]. In Spain, to date, the work by González Domínguez et al. [24] is the only previous study that estimated the savings due to biosimilars. They reported realized savings of EUR 478 million retrospectively from 2009 to 2016 and potential savings of EUR 1965 million in the period 2017 to 2020.

**Figure 1:**
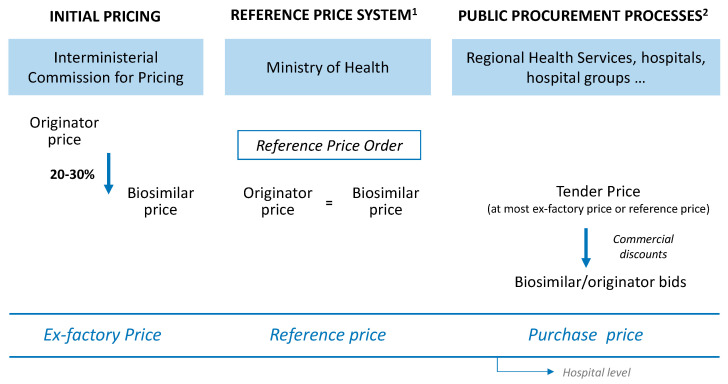
Price regulation of biosimilar medicines in Spain. ^1^ According to Royal Decree 177/2014, of 21 March, regulating the reference price system and the system of homogeneous groups of medicinal products in the National Health System. ^2^ According to Law 9/2017 of 8 November on public sector contracts.

In order to understand the role of commercial discounts in price competition, it is convenient to first look at the regulation of prices for medicines in Spain (Figure 1).

After marketing authorization has been granted, the price for a biosimilar medicine is set by the Interministerial Committee on Pricing of Medicines (ICPM). In general, the ex-factory price (EFP) for the first biosimilar of a given molecule is set at around 20–30% less than the originator price [25]. The same EFP is applied to any other biosimilar of the same molecule that is authorised afterwards. Thus, the originator and biosimilar(s) have different prices for a period of time, not more than one year, since in Spain the price of off-patent medicines is regulated by the Reference Price System (RPS) [26] in the same way as in other European countries [27]. Thus, annually a Reference Price Order (RPO), published in the Official State Gazette (Spanish: Boletín Oficial del Estado, BOE), establishes the reference groups (the reference group is the basic unit of the RPS and it is constituted by at least one presentation of a biosimilar medicinal product that has the same active pharmaceutical ingredient and identical administration route) and fixes the reference price (RP) or the maximum amount of public reimbursement of the presentations of medicinal products included in the reference groups established. After the publication of the corresponding RPO, the biosimilar and originator of a particular molecule share the same RP.

EFP, or RP where applicable, is a fixed price in retail pharmacies (to which pharmacy and distributor margins and VAT are added). However, in Spain most originators and their biosimilars are dispensed at hospital pharmacies (11 of the 16 molecules with biosimilars on the Spanish market). In fact, the percentage of pharmaceutical spending on biosimilars within hospital pharmacy spending has grown continuously, from 1% in 2014 to 3% in 2018 [28]. This fact is relevant because these medicines are mainly purchased via public tenders (currently according to Law 9/2017, of 8 November, on Public Sector Contracts [29]; although previously according to Royal Legislative Decree 3/2011 [30] and before that, accordingly to Law 30/2007, of 30 October, on Public Sector Contracts [31]). Under public procurement, hospitals (or other health providers) tender a contract for the acquisition of medicines (originator and biosimilar) for a determined period and an estimated volume. Then, drug manufactures submit their bids (with a price lower or equal to the price tendered by the entity). The award of the contract depends on the economic offer, although other technical criteria are also taken into consideration. Hence, there is a variable difference between purchase price and EFP price or RP, where applicable, hereinafter referred to as “commercial discount”.

This paper provides a new estimate of the budget impact generated by biosimilars in the National Health System for the years 2009 to 2019. It differs from a previous study in Spain [24] because we take into account the real acquisition scenario, that is, EFP and commercial discounts. This work is part of a wider research project analyzing the budget impact of biosimilars in the Spanish NHS, which published a report (grey literature, not indexed) in Spanish on 27th November 2020 [32].

## 2. Results

According to our results, the budget impact derived from the introduction of biosimilar medicines in the Spanish NHS would reach more than EUR 2306.48 million of cumulative savings in the 11-year period from 2009 to 2019 (Table 1). Somatropin (EUR 375 million), epoetin (EUR 589 million) and infliximab (EUR 450 million) biosimilars provide the greatest contribution to the aggregate savings up to 2019, which is attributable to their long presence on European pharmaceutical market (10, 10 and 5 years, respectively) and the combination of their uptake and price volumes.

**Figure 2:**
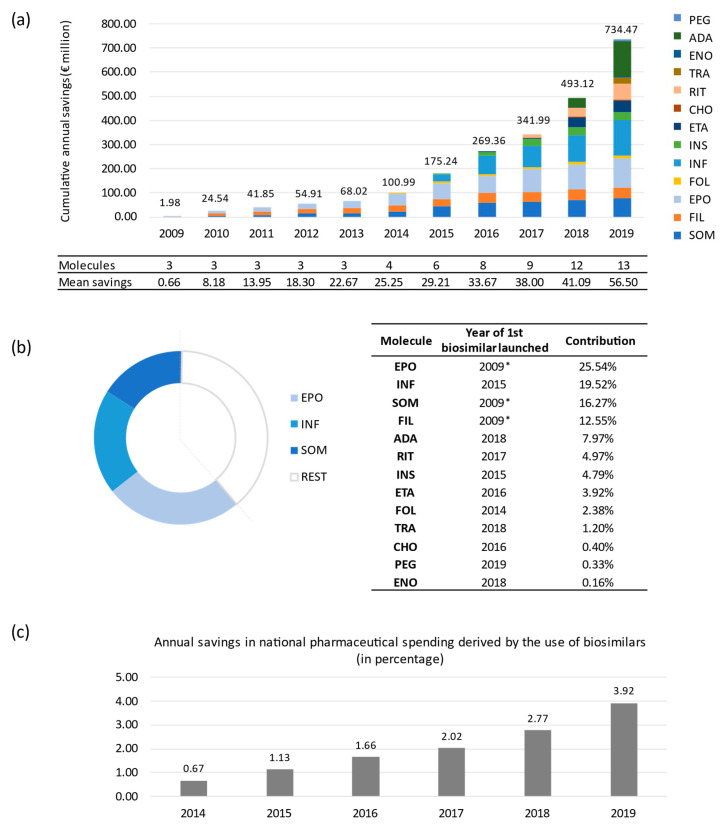
Distribution of aggregate savings. (**a**) Aggregate savings over time. All molecules exposed to biosimilar competition each year were included in the analysis.; (**b**) Specific contribution of each of the molecules to the total amount of savings. SOM: somatropin; FIL: filgrastim; EPO: epoetin; FOL: follitropin alfa; INF: infliximab; INS: insuline glargine; ETA: etanercept; CHO: chondroitin sulfate; RIT: rituximab; TRA: trastuzumab; ENO: sodium enoxaparin; ADA: adalimumab; PEG: pegfilgrastim. * Biosimilars marketed before 2009: somatropin, 2006; filgrastim and epoetin, 2008 (2009 is the first year with available consumption data). (**c**) Annual savings derived from the use of biosimilars, in percentages, since 2014 with respect to total pharmaceutical spending in Spain. Total pharmaceutical spending calculated by adding hospital pharmaceutical spending and spending on pharmaceuticals and medical devices per prescription, all calculated at ex-factory prices [34].

Figure 2a shows the aggregated savings over time and the annual mean savings per effectively marketed biosimilar molecule. The temporal evolution analysis showed a growing trend as more biosimilar medicines enter the market. The breakdown of the contribution by molecule (see Figure 2b) revealed that epoetin is in first position with savings of EUR 589 million, followed by infliximab (EUR 450 million) and somatropin (EUR 375 million). Taken together, these three molecules account for more than 60% of total savings in the entire period. However, the entry of biosimilars of different molecules significantly changes the market and the estimation of savings. For instance, adalimumab ranks fifth (EUR 183.77 million) in just two years in the market and a biosimilar uptake of only 18% in 2019. It is not surprising as it is the most consumed drug in the Spanish NHS in terms of hospital pharmaceutical expenditure [33]. It is also worth noting that the savings derived from the use of biosimilars are starting to account for a significant percentage of pharmaceutical spending in Spain. Figure 2c shows the percentage of annual savings caused by the use of biosimilars with respect to total pharmaceutical spending published by the Ministry of Finance in Spain [34] since 2014, calculated at ex-factory prices (EFP), without discounts. Annual savings increase from 0.67% in 2014 to 3.92% in 2019. In total, from 2014 to 2019, the savings account for 2.11% of total pharmaceutical spending.

### Sensitivity Analysis

**Figure 3:**
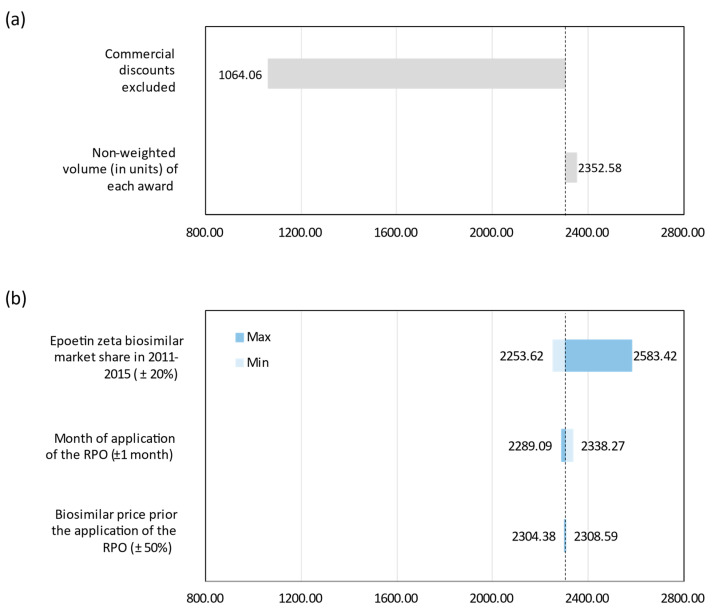
Results of the scenario and sensitivity analyses. (**a**) Scenario analysis. (**b**) One-way sensitivity analysis. The dotted line represents the base case value.

The results from the analysis of alternative scenarios (see Figure 3a) show the great influence of commercial discounts at the hospital tendering on total savings. Significantly, when commercial discounts are excluded, savings realized would be reduced to EUR 1064 million from 2009 to 2019, which means about 50% reduction over the base case results. This scenario would represent the minimum savings due to biosimilar competition (application of the same PR to originator and biosimilar). The other scenario analyzed shows no significant differences from the base case estimate. The same goes for the one-way sensitivity analyses (see Figure 3b). Only the assumptions made in the absence of data (epoetin) show some relevant impact on the savings obtained in the base case, as they affect the data series of two active ingredients whose biosimilars have been on the market for a long time.

Finally, probabilistic sensitivity analysis provides 11-year (2009–2019) cumulative savings with an average of EUR 2310 million (95% IC: EUR 2170–EUR 2461 million) for the NHS. Overall, these results are in line with those obtained for the base case as shown in Table 2.

**Figure 4:**
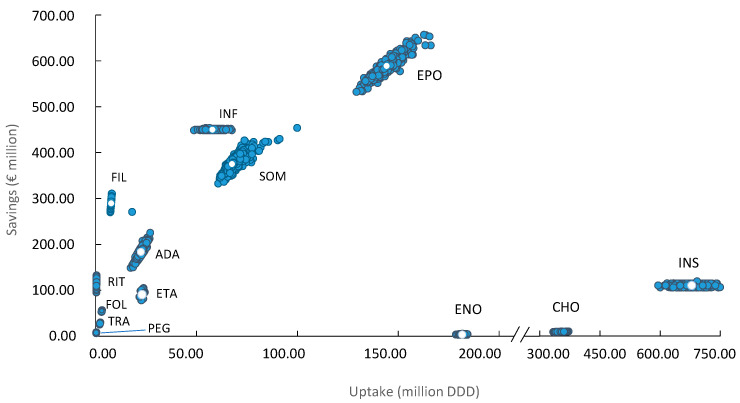
Scatter plot of 1000 Monte Carlo simulations. Vertical axis represents aggregated saving (€ million) for each molecule in the period 2009–2019. Horizontal axis represents the total amount of DDD (million) consumed in this period for each molecule. White dots represent the base case values. SOM: somatropin; FIL: filgrastim; EPO: epoetin; FOL: follitropin alfa; INF: infliximab; INS: insuline glargine; ETA: etanercept; CHO: chondroitin sulfate; RIT: rituximab; TRA: trastuzumab; ENO: sodium enoxaparin; ADA: adalimumab; PEG: pegfilgrastim.

Figure 4 shows the 1000 Monte Carlo simulations performed in the probabilistic analysis. Each of the points represents one of the 1000 simulations carried out. Thus, a greater dispersion of the points along the axes represents a greater uncertainty of the results. As observed, a higher consumption of DDD does not always translate into higher savings, as seen with enoxaparin, chondroitin sulfate and insulin glargine. On the other hand, we see how rituximab achieves considerable savings without reaching high consumption values (in DDD).

## 3. Discussion

To our knowledge, ours is the first study that uses a BIA to estimate the retrospective savings in a European health system for the total of biosimilar molecules marketed and taking into account the real net price (EFP and commercial discounts in the hospital tenders). The only precedent for Spain is González-Domínguez et al. [24]. They estimated the savings derived from biosimilars in the NHS for the retrospective period 2009–2016 and for the prospective period 2017–2020. In order to compare our results to theirs, we have estimated the savings through the budget impact analysis according to our model for the same seven active substances (somatropin, filgrastim, epoetin, follitropin, insulin glargine, infliximab and etanercept) and in the same period (2009–2016). We estimate savings of EUR 343 million compared to EUR 478 million reported by [24]. This difference may be due to different assumptions on the price erosion, the application date of the RPO, or the estimated uptake of each molecule used and merely reflects the complexity inherent to any estimation of savings.

Few studies have calculated the real retrospective savings derived from the introduction of biosimilars in the European context. Simoens et al. [16] reviewed full publications and posters focusing on BIA of biosimilar medicines. Their work revealed the lack of peer-reviewed information on the budget impact of biosimilar products. Only three studies were considered full budget impact models according to ISPOR good practice guidelines. They all aimed to estimate the budget impact of the introduction of an infliximab biosimilar over a prospective time horizon between 1 and 3 years, also considering some type of substitution or combination.

Since then, additional BIAs of biosimilar medicines in Europe have been published. They mainly aimed to analyze the budget impact of one molecule (antiTNF class is the wider class analyzed) in a time horizon between 3 and 5 years. For instance, in Italy, Rognoni et al. [17] estimates the impact of the use of a rituximab biosimilar in the Italian National Health System in a 3- and 5-year horizon that accounts for EUR 79.2 and EUR 153.6 million, respectively. Likewise, the introduction of an adalimumab biosimilar would generate savings of EUR 260 million in 5 years [19]. In the United Kingdom, Aladul et al. [20] updated their previous study [35] including the introduction of a new antiTNF biosimilar in the areas of rheumatology and gastroenterology. According to their calculations the impact would amount, in a 3-year horizon, to GBP 285 million. Other studies expand the focus to EU5 (infliximab) [36] or a greater pool of European countries (rituximab) [37]. In a very recent study, Agirrezabal et al. [21] estimated the impact of biosimilar insulin glargine in primary care in the NHS with, specifically, savings of GBP 900,000 between October 2015 and December 2018. They also provide an estimate of the savings lost due to reduced use of biosimilars, which could have reached GBP 25.6 million, indicating that only 3.42% of the potential savings have been achieved.

Of note, most studies cited used ex-factory prices excluding discounts as cost-input. This does not reflect reality, as hospitals usually negotiate individual discounts through public tenders. By contrast, our study uses purchase prices paid by hospitals. We believe this allows for a more accurate estimation of savings due to biosimilar competition in Spain since 2009. At the same time, the large period of time we analyze, from 2009 to 2019, allows us to observe how, in general and per molecule, savings are increasing in time.

In any case, our results are broadly consistent with the observed financial impacts from other countries in that biosimilar uptake translates into significant savings and that when longer periods are considered, higher savings are realized, as expected.

It is important to note that the estimated savings are affected by the variation and level of both quantities and prices. Consequently, a higher consumption of DDD does not always translate into higher savings, as observed with enoxaparin, chondroitin sulfate and insulin glargine, due to a lower price with respect to complex biosimilar molecules such as antiTNF or monoclonal antibodies. For the same reason, rituximab achieves considerable savings without reaching high consumption values.

It is worthwhile to highlight that adalimumab (the first biosimilar launched at the end of 2018) accounts for almost 8% of the 11-year (2009–2019) cumulative savings. This figure corresponds to realized savings of EUR 187 million in scarcely one year. It is not surprising as adalimumab is responsible for the highest drug spending in Spain [33]. This suggests that higher savings in the short term may be expected. In fact, we have estimated that the percentage of annual savings caused by the use of biosimilars with respect to total pharmaceutical spending is increasing and by 2019 it was 3.92%.

An additional finding of this work is that potential savings in Spain due to biosimilars are not yet fully exploited, as the biosimilar uptake is still lower than that in other countries, at least for some active substances. For example, antiTNF biosimilar uptake in Spain was 49% in 2019 vs. Denmark (96%), Germany (61%), Italy (64%) or Norway (74%). The same pattern is observed for biosimilar monoclonal antibodies in oncology. The penetration in Spanish market barely exceeds 35% vs. Denmark (74%), Germany (49%), Italy (52%) or Norway (70%). This lower utilization proves that there is a room for improvement in the Spanish NHS [38].

In any case, it is important to note that different molecules behave differently and not all contribute equally to savings in each country, or in different countries, because of the different price and reimbursement policies, procurement procedures, and other pharmaceutical policies, which vary greatly among European countries.

In the case of Spain, a comprehensive report by the Independent Authority for Fiscal Responsibility [39] confirmed the high variability across autonomous communities in terms of uptake levels and promotion policies. This may have been a driver for the Ministry of Health’s attempt to establish a national policy on biosimilars [40]. This plan, still under revision, makes recommendations to revisit those supply and demand policies put in place in Spain with the further aim of promoting the utilization of biosimilar medicines in Spain. This aim is also supported by the Advisory Committee for the Funding of the Pharmaceutical Benefit of the National Health System, a collegiate body attached to the Ministry of Health. In its analysis of this plan, the committee is of the opinion that promoting the use of biosimilars will lead to more competition and reduction of the burden of pharmaceutical spending [41]. We consider that more research is needed on the role of biosimilar competition in pharmaceutical cost containment. Given the increasing concern regarding the sustainability of healthcare systems, and the contribution biosimilars can play towards that end, in line with our findings, more ambitious or fine-tuned policies for promoting biosimilars (in general or some biosimilars specifically) may be expected.

### Limitations

As in any other study, this retrospective BIA has certain limitations, mainly due to the non-availability of data, specifically among the first three biosimilar classes on the market (EPO, G-CSF, and hGH). Additional sources [42,43,44] were used to complete information gaps on the uptake of these biosimilars. When the price of the biosimilar prior the RPO launch was unknown, we assumed that it was a 10% higher than the price after RPO. We believe this assumption is a conservative position, as the RPO can lead to price reductions of up to 30%, as in the case of adalimumab.

Regarding the estimation of commercial discounts, as mentioned, a sample of 143 public procurement tenders (the most recent in each autonomous community) was used. Although the sample was considered to be representative, it does not include all the public procurement procedures in the country for the entire period of analysis. This is because sharing transparent information on purchase prices (tenders) is a very recent trend motivated by the EU directives on public procurement. In addition, we acknowledge that an unequal distribution of tender procedures per region might influence the estimation of actual savings in Spain. The degree of variability in the level of discounts awarded via public tenders for the same molecule within the regions is out of the scope of this research and merits itself further exploration. In addition, to overcome the lack of data on the public tenders prior to 2016, a linear regression was performed with 0% as the lower limit of discount matching the time of biosimilar launch. This would represent the evolution of price discounts derived from competition between an originator and biosimilars over the years.

The results of this BIA should be interpreted with these limitations in mind.

## 4. Materials and Methods

### 4.1. Model Design

**Figure 5:**
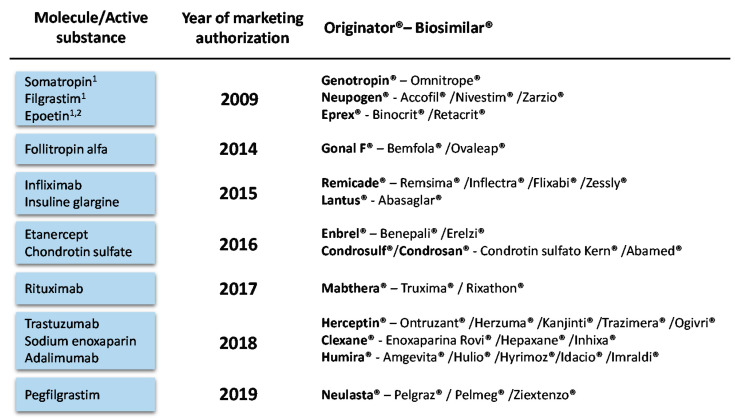
Biosimilar medicines effectively marketed in Spain as of December 2019. ^1^ Biosimilars marketed before 2009 (2009 is the first year with available consumption data). ^2^ For the purpose of this study, epoetin zeta and alfa are considered as a single molecule. Bevacizumab and teriparatide biosimilars have been recently marketed in Spain (September 2019 and June 2020, respectively) but they are not included in this analysis.

We perform a retrospective BIA of the introduction of biosimilars from the Spanish Health System perspective covering the period from 2009 to 2019. All the molecules exposed to biosimilar competition in this period were included in the analysis (Figure 5). We adopt the third-party payer perspective and thus, we only account for direct medical costs, in particular pharmacological costs prior to and after the market introduction of biosimilars. The calculation was conducted in a Microsoft Excel-based spreadsheet model. The model was constructed in compliance with methodology guidelines for economic evaluations and analyses previously developed in Spain [45,46].

To estimate the budget impact, two scenarios are compared. First, the hypothetical scenario in which biosimilar drugs are not available on the market and therefore, an originator’s price would keep constant throughout the period examined. This assumption is based on a review of the price evolution of originators (anti-TNFs, trastuzumab and rituximab). We found that these originator medicines did not undergo major price changes before biosimilar entry. This could be interpreted to mean that even if other molecules generate competition in the same indication, the originator’s price is rarely modified. Second, the actual scenario with biosimilars available on the market after an originator’s patent expiration is examined. In this scenario, competition leads to a price reduction for originator medicines. The difference in terms of costs between the two scenarios provides the savings generated by the introduction of biosimilars.

The two main variables of the analysis are uptake (consumption data) and price for each molecule, both originator and biosimilar. To provide clarity on some specific terminology a glossary table (see Table 3) with English terms used in this manuscript and their Spanish equivalents is provided.

### 4.2. Uptake Estimation

**Figure 6:**
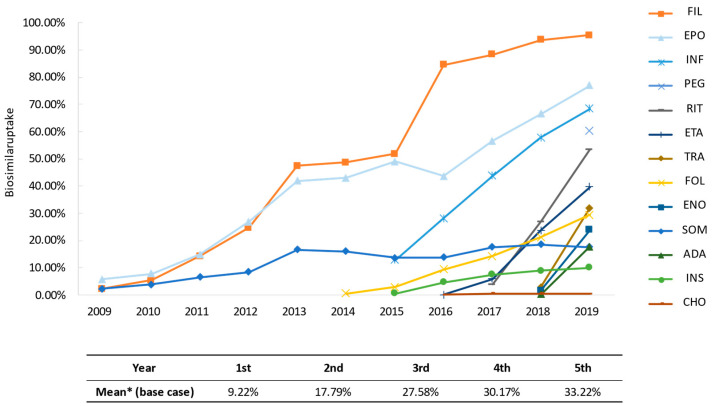
Biosimilar penetration in Spain over time (adapted from [29]). Biosimilar uptake (%) is calculated as volume of biosimilars over volume of biosimilars plus the originator product (DDDs). * Chondroitin sulfate and the three biosimilars marketed before 2009 (somatropin, filgrastim and epoetin) were excluded to avoid distorting the mean. SOM: somatropin; FIL: filgrastim; EPO: epoetin; FOL: follitropin alfa; INF: infliximab; INS: insuline glargine; ETA: etanercept; CHO: chondroitin sulfate; RIT: rituximab; TRA: trastuzumab; ENO: sodium enoxaparin; ADA: adalimumab; PEG: pegfilgrastim.

We use two sources of data on the uptake of biosimilars. For the period between 2009 and 2015, we use data provided by all manufacturers, representing the number of units effectively consumed by the NHS (BioSim, data on file) and for the period between 2016 and 2019, we use data provided by the Ministry of Health (Ministry of Health, data on file). In both scenarios (with and without biosimilars) the volumes have been converted to daily doses using the published World Health Organization (WHO) defined daily doses (DDD) [47] as previously used by Haustein et al. [48] to estimate the impact of the introduction of biosimilars in several European countries. Figure 6 shows the evolution over time of biosimilar uptake in the Spanish pharmaceutical market. In the bottom of the figure, the estimated average uptake after launch of the first biosimilar (that means, mean uptake of first year of commercialization, mean uptake of second year of commercialization, and so on) are shown.

### 4.3. Price Estimation

For each molecule, drug acquisition costs (EFP) for the year 2019 have been obtained from BotPlus, the Health Information database of the General Council of the Association of Official Pharmacists that provides harmonized information on medicines [49]. For the previous years, price evolution was also obtained from BotPlus, and when not available, these prices were provided by BioSim (BioSim, data on file). RPO published in the OSG from 2014 to 2019 were consulted to provide RP. RP is assumed to affect price calculations in the same month of the publication of the RPO when it is prior to the 15th day of the month, otherwise RP will apply the following month. When a biosimilar price between its commercialization and its regulation by the reference pricing system is unknown, we assume an increase of 10% over the RP, following the observation of other biosimilars for which full price data are available.

Purchase prices in hospital tenders were used to calculate discounted prices per DDD compared to the EFP for infliximab, etanercept, adalimumab, trastuzumab and rituximab (data from 143 public tenders collected by Acobur S.L. (https://www.acobur.es, accessed on 15 April 2020). This reduced price was weighted by the total volume (in units) of each award (of originator and biosimilars) to obtain the commercial discount for each molecule and year. In the case of somatropin, epoetin, filgrastim and pegfilgrastim, internal BioSim data for years 2018 and 2019 were used and a linear regression was conducted assuming a discount of 0% the year before the marketing of the first biosimilar. No discount is considered for follitropin alfa, insulin glargine, chondroitin sulfate and enoxaparin, as they are mostly dispensed by retail pharmacies, where commercial discounts do not apply. Table 4 shows the number of tenders analyzed and the level of discount for each molecule.

### 4.4. Molecule-Specific Assumptions

In addition to the general assumptions mentioned above, it was necessary to adopt other specific assumptions given the lack of specific information about both uptake and price variables (Table S1).

### 4.5. Sensitivity Analsyses

In order to evaluate the uncertainty associated with the variables used in the budget impact model and determine the robustness of the results obtained, we carried out both deterministic and probabilistic sensitivity analyses.

In the scenario analysis, some of the assumptions are modified with respect to the base case (non-additively) (Table 5). The new alternatives (non-additive) propose different ways to calculate the price variable. Scenario 1 estimates the impact on price of ignoring the discounts that manufacturers give to hospitals. Scenario 2 ignores the volume-weighting, that is to say, the purchase price only applies to the year in which the tender is awarded regardless of the duration of the contract.

One-way sensitivity analysis was performed by changing, one by one, some parameters of the model: the price of some biosimilars prior the application of the RPO, the month of application of the RPO, and the market share of biosimilar epoetin in 2011–2015 (data from [42]) (Table 5).

We also performed a probabilistic sensitivity analysis using the Monte Carlo method with 1000 simulations, simultaneously modifying all parameters from base-case values following a normal distribution, in line with the recommendations of the literature [50].

## 5. Conclusions

The increase of health expenditures, and in particular, of pharmaceutical expenditures in Spain highlights the need for effective strategies to contain and rationalize pharmaceutical spending.

This is the first study carried out which jointly analyzes the savings for the Spanish NHS in terms of pharmaceutical expenditure derived from both the uptake of biosimilar products and the downward effect on prices resulting from competition (RPS and public tenders, with commercial discounts).

Our results show how the introduction of biosimilar drugs in the Spanish pharmaceutical market has brought competition in the market of biological products, and unquestionable, increasing and significant savings, especially at the hospital level, where the majority prescriptions for the molecules herein analyzed are issued. Thus, biosimilar medicines represent a great opportunity to promote the sustainability of the NHS through rationalization and efficiency in pharmaceutical expenditure. Our study also shows that the full potential of biosimilar savings has not been achieved yet, as there is a high variability in biosimilar uptake across autonomous regions.

This is a first approach to the impact of biosimilar medicines on the pharmaceutical market in terms of price competition, uptake and savings. However, a further research might address other issues such as level of competition, variability across molecules and within regions, relationships, if any, between market size and number of competitors or price discounts.

In any case, any pharmaceutical policy to be adopted should not only analyze its expected impact in the short-term, but also in the medium- and long-term, to promote healthy competition in the market for biological pharmaceutical products, whether originator or biosimilar. After all, the ultimate goal is the sustainability of the healthcare system with rapid access to innovative products, but also a healthy competition from biosimilars when the patent from the originators expires, resulting in better access for patients to obtain the clinical benefits derived from the treatments.

## Figures and Tables

**Table 1:** Results of the BIA (€ million savings).

**Molecule ^1^**	**Scenario without Biosimilars**	**Scenario with Biosimilars**	**Realized Savings**
**SOM**	992.5	617.32	375.18
**FIL**	469.62	180.22	289.4
**EPO**	993.01	403.86	589.15
**FOL**	119.2	64.27	54.92
**INF**	1054.47	604.18	450.3
**INS**	818.41	707.87	110.54
**ETA**	628.39	537.91	90.48
**CHO**	134.67	125.34	9.32
**RIT**	433.39	318.76	114.62
**TRA**	140.89	113.26	27.63
**ENO**	264.86	261.25	3.62
**ADA**	772.46	588.69	183.77
**PEG**	10.77	3.2	7.57
**TOTAL**	6832.63	4526.15	2306.48

^1^ SOM: somatropin; FIL: filgrastim; EPO: epoetin; FOL: follitropin alfa; INF: infliximab; INS: insuline glargine; ETA: etanercept; CHO: chondroitin sulfate; RIT: rituximab; TRA: trastuzumab; ENO: sodium enoxaparin; ADA: adalimumab; PEG: pegfilgrastim.

**Table 2:** Results of the probabilistic sensitivity analysis (€ million).

**Molecule ^1^**	**Base Case**	**Probabilistic Sensitivity Analysis**			
		**Mean**	**95% CI**		
**SOM**	375.18	377.96	348.25	-	415.67
**FIL**	289.40	289.27	274.55	-	303.17
**EPO**	589.15	590.26	546.85	-	634.32
**FOL**	54.92	54.92	53.71	-	56.07
**INF**	450.30	450.25	448.59	-	452.03
**INS**	110.54	110.40	106.08	-	115.03
**ETA**	90.48	90.60	83.26	-	99.25
**CHO**	9.32	9.32	8.99	-	9.68
**RIT**	114.62	114.63	103.36	-	126.12
**TRA**	27.63	27.61	26.24	-	29.04
**ENO**	3.62	3.61	2.27	-	4.83
**ADA**	183.77	184.08	161.25	-	207.42
**PEG**	7.57	7.57	6.79	-	8.32
**TOTAL**	2306.48	2310.47	2170.19	-	2460.96

^1^ SOM: somatropin; FIL: filgrastim; EPO: epoetin; FOL: follitropin alfa; INF: infliximab; INS: insuline glargine; ETA: etanercept; CHO: chondroitin sulfate; RIT: rituximab; TRA: trastuzumab; ENO: sodium enoxaparin; ADA: adalimumab; PEG: pegfilgrastim.

**Table 3:** Glossary of English/Spanish terms and their abbreviations.

**English Term**	**Spanish Term**	**Abbreviation in Spanish**
National Health System (abbreviated in text as NHS)	Sistema Nacional de Salud	SNS
Interministerial Committee on Pricing of Medicines (abbreviated in text as ICPM)	Comisión Interministerial de Precios de Medicamentos	CIPM
Ex-factory price (abbreviated in text as EFP)	Precio de venta del laboratorio	PVL
Reference price (abbreviated in text as RP)	Precio de referencia	PR
Reference Price Order (abbreviated in text as RPO)	Orden de Precios de Referencia	OPR
Reference Price System (abbreviated in text as RPS)	Sistema de Precios de Referencia	SPR
Purchase price	Precio de adquisición	-
Official State Gazette (abbreviated in text as OSG)	Boletín Oficial del Estado	BOE

**Table 4:** Level of discount on price per molecule.

**Molecule ^1^**	**Year of First Biosimilar Launch**	**Public Tenders Analyzed ^3^**	**Current Level of Discount**	
			**Original**	**Biosimilar**
**SOM**	2009 ^2^	9	++	++
**FIL**	2009 ^2^	2	+++	+++
**EPO**	2009 ^2^	2	+++	+++
**FOL**	2014	-	-	-
**INF**	2015	37	+	+++
**INS**	2015	-	-	-
**ETA**	2016	55	+	++
**CHO**	2016	-	-	-
**RIT**	2017	36	+	++
**TRA**	2018	21	+	+++
**ENO**	2018	-	-	-
**ADA**	2018	59	+	+++
**PEG**	2019	2	+++	+++

Level of discount on price (either EFP or RP): + (low) 0–25%; ++ (medium) 25–50%; +++ (high) >50%. ^1^ SOM: somatropin; FIL: filgrastim; EPO: epoetin; FOL: follitropin alfa; INF: infliximab; INS: insuline glargine; ETA: etanercept; CHO: chondroitin sulfate; RIT: rituximab; TRA: trastuzumab; ENO: sodium enoxaparin; ADA: adalimumab; PEG: pegfilgrastim; ^2^ Biosimilars marketed before 2009: somatropin, 2006; filgrastim and epoetin, 2008 (2009 is the first year with available consumption data). ^3^ The amount of public tender analyzed exceed 143 as some of them were tendered for several molecules.

**Table 5:** Scenario and one-way sensitivity analyses.

**Scenario**	**Parameter**	**Variation with Respect to the Base Case**
**Scenario 1**	Originator and biosimilar prices	No commercial discounts applied
**Scenario 2**	Commercial discounts (tenders)	No volume weighting applied
**One-way**	**Parameter**	**Variation with Respect to the Base Case**
**One-way 1**	Biosimilar price prior RPO	±50%
**One-way 2**	Month of application of RPO	±1 month
**One-way 3**	Epoetin zeta market share distribution	±20%

## Data Availability

Data is contained within the article.

## Supplementary Materials

The following are available online at https://www.mdpi.com/article/10.3390/ph14040348/s1, Table S1: Assumptions adopted for each molecule.
