# Supplementary material for: Budget Impact Analysis of Biosimilar Products in Spain in the Period 2009–2019
Source: Pharmaceuticals (Basel). 2021 Apr 9;14(4):348. doi: 10.3390/ph14040348 (PMC8069914; doi:10.3390/ph14040348)
Supplement: Supplementary file 1 [file pharmaceuticals-14-00348-s001.pdf]

pharmaceuticals

Table S1. Assumptions adopted for each molecule.

Molecule[1]	Uptake		Price	
	Missing data	Assumption	Missing data	Assumption
**SOM**	Data on originator uptake between 2009 and 2015	-Biosimilar market share between 2011 and 2015 from Almarza et al., [42].<br>- Biosimilar market share in 2009 from Rovira et al. [43].	Biosimilar price prior to RPO-2014	Biosimilar price prior to RPO-2014 is 10% greater than the RP according RPO-2014
**FIL**	Full data available		Biosimilar price prior to RPO-2014	Biosimilar price prior to RPO-2014 is 10% greater than the RP according RPO-2014
**EPO**	- Data on originator uptake between 2009 and 2015<br>- Data on epoetin zeta biosimilar uptake between 2009 and 2015	-Biosimilar market share between 2011 and 2015 from Almarza et al., [42].<br>- Biosimilar market share in 2009 from Rovira et al. [43]<br>- The annual variation is assumed to be proportional to the total uptake of the molecule	Biosimilar price prior to RPO-2014	Biosimilar price prior to RPO-2014 is 10% greater than the RP according RPO-2014
**FOL**	Data on originator and biosimilar uptake between 2014 and 2016	- Biosimilar market share in 2015 extracted from Comunitat Valenciana, [44].<br>- The total volume of the molecule in 2015 is taken as the total market in 2014. Biosimilar, marketed in November 2014, is assumed to capture 20% of 2015 market.	Biosimilar price prior to RPO-2015	Biosimilar price prior to RPO-2015 is 10% greater than the RP according RPO-2015
**INF**	Full data available		Full data available	
**INS**	Full data available		Biosimilar price prior to RPO-2016	Biosimilar price prior to RPO-2016 is 10% greater than the RP according to RPO-2016
**ETA**	Full data available		Full data available	
**CHO**	Full data available		Biosimilar price prior to RPO-2017	Biosimilar price prior to RPO-2017 is 10% greater than the RP according RPO-2017
**RIT**	Full data available		Full data available	
**TRA**	Full data available		Full data available	
**ENO**	Full data available		Biosimilar price prior to RPO-2019	Biosimilar price prior to RPO-2019 is 10% greater than the RP according RPO-2019
**ADA**	Full data available		Full data available	
**PEG**	Full data available		Full data available	

[1] SOM: somatropin; FIL: Filgrastim; EPO: epoetin; FOL: follitropin alfa; INF: infliximab; INS: insuline glargine; ETA: etancercept; CHO: chondroitin sulfate; RIT: rituximab; TRA: trastuzumab; ENO: sodium enoxaparin; ADA: adalimumab; PEG: pegfilgrastim.
